# Health economic evaluations of diagnostic tests for tuberculosis: a narrative review

**DOI:** 10.1186/s13561-025-00639-2

**Published:** 2025-05-24

**Authors:** Cemre Arpa, Ahmed Abd El Wahed, Serap Aydin, Prakash Ghosh, Dinesh Mondal, Lydia Nakiyingi, Julius Boniface Okuni, Sophie Schneitler, Manfred Weidmann, Martin Siegel

**Affiliations:** 1 Department of Empirical Health Economics, Technische Universität Berlin, Berlin, Germany; 2 Heidelberg Institute of Global Health, Heidelberg University, Heidelberg, Germany; 3 Institute for Animal Hygiene and Veterinary Public Health, Leipzig University, Leipzig, Germany; 4 International Centre for Diarrheal Disease Research Bangladesh (icddr,b), Dhaka, Bangladesh; 5 Makerere University College of Health Sciences, Kampala, Uganda; 6 College of Veterinary Medicine, Animal Resources and Biosecurity, Makerere University, Kampala, Uganda; 7 Institute of Medical Microbiology and Hygiene, University Clinic Saarland, Homburg (Saar), Germany; 8 Midge Medical GmbH, Berlin, Germany

**Keywords:** Tuberculosis, Diagnostics, Health economics, Evaluation, Reporting, Guidelines, Transparency

## Abstract

**Background:**

Tuberculosis is the leading cause of death from infectious diseases globally. Non-specific symptoms and limitations of existing diagnostics involve challenges for informed policymaking and clinical practice. This paper reviews common practices in reporting the selection and definition of cost and effect parameters, and in reporting the translation of effect parameters into utility and disability weights, in health economic evaluations of TB diagnostic tests.

**Methods:**

A targeted literature search in PubMed, Cochrane Library, Web of Science, and Google Scholar identified health economic evaluations of diagnosis and population screening strategies for TB.

**Results:**

We found 28 studies comprising 11 cost-effectiveness and 17 cost-utility analyses. Observed patient data were used in 6 studies, 22 relied solely on model-based evaluations. Variations in prevalence, accuracy, and utility parameters were common, the Consolidated Health Economic Evaluation Reporting Standards terminology for costing was only used in 8 out of 28 studies.

**Discussion:**

Future studies should state the exact type of TB studied, as it can manifest in multiple organs, remain inactive for long periods of time, and since different diagnostics can perform differently depending on the site involved it may influence test accuracies. Additionally, potential impacts of sequential diagnostics on test accuracy and the cost of inaction should receive more attention.

**Conclusion:**

Precise terminology and transparent definitions of parameters and methodology in health economics evaluations are necessary to generate evidence that guides policymakers and supports clinical decision-making in the context of TB.

**Supplementary Information:**

The online version contains supplementary material available at 10.1186/s13561-025-00639-2.

## Introduction

With 1.25 million deaths in 2023, tuberculosis (TB) returns to be the deadliest infectious disease, surpassing COVID-19, and causing almost twice as many deaths as HIV/AIDS [1]. The World Health Organization (WHO) set the ambitious goal to reduce TB incidence by 90% by 2035, compared to 2015 levels, through the “End TB Strategy”, which ultimately aims to eliminate the disease [2]. While TB incidence rates in high-income countries are generally low and declining, low- and middle-income countries continue to face significant challenges with TB. The 30 countries with the highest TB-related disease burden bear 87% of all tuberculosis cases reported in 2023, and only five countries (India, Indonesia, China, the Philippines, and Pakistan) accounted for 56% of all TB cases in 2023 [1].

The disproportionate distribution of TB cases, particularly in low- and middle-income countries, highlights the need for strategic resource allocation to combat its spread and impact effectively. Health economic evaluations, defined by Drummond et al. as a comparative analysis of alternative courses of action in terms of both their costs and consequences, are a pivotal approach for generating evidence to inform and guide policymakers. These can be distinguished into cost-effectiveness-analysis where the outcome is measured as clinical parameters in natural units in a single dimension, cost-utility-analysis where multi-dimensional utility-based outcomes are used, and cost-benefit-analysis, where preferences are used to convert an effect into monetary units. All three measure the costs in monetary units, and all aim to identify cost-effective strategies for decision making [3]. To enable informed decisions, ensuring high reporting standards in these evaluations is essential when comparing different evaluations across time, countries, and technologies.

Mostly, transmission of *Mycobacterium tuberculosis* (MTB) occurs through aerosols containing viable pathogens transmitted via the respiratory tract [4]. Adequate immune responses prevent active infection in approximately 90% of individuals [5]. Active TB primarily manifests in the lungs as pulmonary TB (PTB) and accounts for 85% of reported TB cases worldwide. In cases where other parts of the body are affected, it is referred to as extrapulmonary TB (EPTB) [6]. Latent TB infections (LTBI), where MTB are present but in a dormant state, pose no immediate risk of transmission, but the risk of developing active TB persists for decades [7].

Selecting an appropriate reference test for TB diagnosis is highly challenging due to the complexity of TB detection. Each diagnostic approach has its strengths and limitations. Tests for TB infection, like the Tuberculin Skin Test (TST) and Interferon-Gamma Release Assays (IGRAs), detect exposure to the bacteria but cannot differentiate between latent and active disease. TST is cheaper and easier to administer, while IGRAs offer greater specificity and are not affected by Bacillus Calmette Guerin vaccinations. To diagnose active TB, tests like Chest X-rays (CXRs), CT scans, and sputum smear microscopy (SSM) are used. CXRs are widely available and inexpensive but lack sensitivity. CT scans provide more detailed images but are costlier, are not readily available in most low-level health centers, and involve higher radiation exposure. SSM offers rapid results but has low sensitivity, especially in HIV-positive patients. Nucleic Acid Amplification Tests (NAATs), such as GeneXpert-PCR MTB/Rif, provide rapid and specific results, including detection of drug resistance, but are more expensive, resource-demanding, and require expert technicians. Culture, the gold standard, offers definitive diagnosis and drug-resistance profiling but is time-consuming and requires sophisticated infrastructure. Finally, Lateral flow immunochromatographic assays (TB-LAM), which detect TB antigens in urine, are rapid and user-friendly, particularly in HIV-positive individuals, but have lower sensitivity overall. Choosing the right test depends on the patient’s individual circumstances and the available resources, often leading to a combination of tests for accurate diagnosis.

This paper emphasizes the critical role of reporting standards in health economic evaluations of TB diagnostics. It reviews common practices in how differently cost and effect parameters are selected, defined, justified, and reported in existing literature, and in how much detail methods for the translation of effect parameters into utility and disability weights are commonly reported. The diverging styles and standards found in this review highlight the importance of describing the common building blocks of TB evaluation studies, such as cost and effect parameters and the test accuracy, transparently and in enough detail to ensure comparability across studies, contexts, and populations. This is particularly important in model-based evaluations of TB diagnostics, as TB can manifest in manifold ways and testing is challenging even under optimal circumstances.

## Methods

### Search strategy

A targeted literature search for economic evaluations in the field of TB diagnostics was conducted using the PubMed, Cochrane Library, and Web of Science databases using the following Boolean operators: („tuberculosis“) AND („screening“), („tuberculo-sis“) AND („cost-eff*“), („tuberculosis“) AND („cost-util*“), („tuberculosis“) AND („cost-benefit“), („tuberculosis“) AND („economic evaluation“) and („tuberculosis“) AND („diagnosis“). In addition, the keywords were also combined with the names of diagnostic tools, such as “Xpert”. Studies were only included in the final assessment if they evaluated the cost-effectiveness of the screening or diagnostic tools. Furthermore, a search was performed on Google Scholar and cross-referenced with the results obtained from the aforementioned databases. Moreover, a snowball search was conducted to identify additional relevant publications. Only English literature was screened, and a temporal restriction was applied, selecting studies published between January 1, 2012, and April 1, 2024. This focused approach allowed the critical assessment of key elements, their reporting practices, and methodological shortcomings in widely published health economic evaluation studies in TB diagnostics, rather than capturing all available studies comprehensively.

### Inclusion and exclusion criteria

Textbox 1 outlines the inclusion and exclusion criteria used in the study selection process for this narrative review. We refrained from using the CHEERS criteria to determine the inclusion or exclusion of the studies discussed below, because the exact results and their reliability were not within the scope of this review. Instead, we focus on providing examples of good and potentially problematic reporting practices in order to provide guidance for researchers in the field.

**Textbox 1:** The inclusion and exclusion criteria for the study selection process

**Inclusion criteria**
• Economic evaluation of TB screening and diagnostic tools
• Explicitly stated reference and index strategy
• E.g., microscopy vs. culture or microscopy vs. no diagnostic
• Published in English language between January 1, 2012, and April 1, 2024
**Exclusion criteria**
• Principal evaluation of a screening strategies (e.g. annual vs. targeted screening)
• Only identifying the necessity for screening in a certain population, without comparisons of diagnostic tools or strategies
• All types of reviews
• Comparison of different versions of the same TB diagnostic tools that solely detect drug resistance

### Data extraction

Systematic data extraction was conducted on the chosen studies, encompassing demographic characteristics of the study population, geographical location, tuberculosis classification, model type, and design specifications, the perspective adopted in the economic evaluation, reference and index test strategies investigated, incremental cost-effectiveness ratio (ICER) alongside relevant cost and utility metrics, discount rate, time horizon, sources for utility and disability weights, and Willingness-to-Pay (WTP) thresholds.

### Cost assessment

We focused on common cost assessment methodologies found in the literature and refrained from elaborating on details of the included cost items in each of the included studies, as these depend on the perspectives, study settings, and project budgets. Instead, we employed the classification from the Consolidated Health Economic Evaluation Reporting Standards (CHEERS) in order to distinguish different practices concerning the valuation and measurement of resources and their underlying data sources [8]. Cost valuation can be done either by top-down approaches, bottom-up approaches, or a combination of the two. Top-down costing allocates expenditures accumulated at each organizational cost center to units of activity. In contrast, bottom-up costing initially identifies the resources utilized by individual patients and then assigns unit costs to these resources to determine the total cost per patient [9]. Measurement describes the level of aggregation in identifying and measuring costs and resources. We denote highly aggregated cost data as gross-costing, and detailed cost structures as micro-costing [9].

### Effect parameters

We identified primary and secondary effect parameters, as well as disability and utility measurements in each included study. Furthermore, we investigated these parameters concerning their definitions and, with regards to utility and disability parameters, their utility and disability weight assessment to identify similarities and discrepancies.

### Classification of analysis types

We classified each health economic evaluation based on its selection of effect parameters. Evaluations were categorized as cost-effectiveness analyses (CEA) if they used clinical parameters, and cost-utility analyses (CUA) if aggregated utility parameters like Quality-Adjusted Life Years (QALYs) and Disability-Adjusted Life Years (DALYs) were used. One may argue that willingness-to-pay (WTP) thresholds imply a societal consensus on the monetary value of the outcome, such that studies employing such WTP thresholds should qualify as cost-benefit analyses. However, we followed the common practice in the literature and only categorized an evaluation as a cost-benefit analysis if stated or revealed preferences were measured.

## Results

### Study characteristics

The search strategy yielded 28 studies, comprising 11 cost-effectiveness analyses and 17 cost-utility analyses, and only one containing no model-based evaluation, while no cost-benefit analyses were found through our search. Among the 27 model-based evaluations, decision tree models were the most commonly used modeling approach (*n* = 17), followed by Markov models (*n* = 8) and microsimulation models (*n* = 2). Populations from more than 21 nations were analyzed. Most studies addressed countries with high TB incidence rates of at least 40 cases per 100,000 citizens, some included multiple countries. Sub-Saharan African countries were included in *n* = 20 studies, Southeast Asian countries were included in *n* = 12 studies.

Only 6 of the 28 studies utilized patient data [10–15], whereas 22 of the 28 studies were simulation studies based on model parameters taken from the published literature. The study populations primarily included adults and individuals with HIV. Additional details about the population characteristics are provided in Table S1 in the appendix. The exact type of TB infection was stated only in 10 out of the 28 studies [10, 15–23], the remaining 18 either used the general term TB or referred to active TB without further specifying the type.

Since the reference diagnostic test is crucial in evaluating an index test, Table 1 gives an overview of TB diagnostic tools, detailing their strengths and weaknesses. A further detailed presentation of all included studies with their respective reference and index tests is provided in Table 2.

**Table 1:** Overview - Strengths and weaknesses of common TB diagnostic tools

Technique	Strength	Weakness	Source
Tuberculin skin test (TST)	• Cheap, easy to use • No elaborate laboratory infrastructure necessary • LTBI detection	• Limited sensitivity and specificity • No active TB detection • Re-presentation of the patient, longer evaluation period • Host genetics may influence test sensitivity	[51–54]
Interferon-Gamma-Release Assay (IGRA like QuantiFERON^®^-TB-Gold)	• Higher specificity than TST • Usually results the following day • LTBI detection	• Specialized laboratory needed • Cost-intensive • No active TB detection • Applicable for children over 5 years • Host genetics may influence test sensitivity	[51–54]
Chest X-ray (CXR)	• Localization of foci of inflammation possible • Inexpensive • Active TB detection	• Diagnosis dependent on expert interpretation • Lower sensitivity than CTs • Not applicable for LTBI • Not effective for EPTB	[5,51,55]
Computer tomography (CT)	• Extent of infection possible • High sensitivity • Active TB detection	• Cost-intensive • Not applicable for LTBI	[56]
Sonography	• Non-Invasive & Radiation-Free • Effective for EPTB	• Limited pulmonary Imaging • Lower sensitivity than CTs • Not applicable for LTBI	[57]
Smear Microscopy (SM)	• Inexpensive, easy to use • Fast results • High specificity in high prevalence TB-populations • Active TB detection	• Varying sensitivity • Not applicable for LTBI • Less sensitive in children • Multiple visits necessary	[2,5,58,59]
NAAT (PCR) e.g. GeneXpert MTB/RIF	• Fast outcome • High specificity • Drug resistance testing for INH/RMP possible • Active TB detection	• Varying sensitivity • Not applicable for LTBI • Trained lab personnel • Expensive	[5,60]
Culture	• High sensitivity & specificity (Gold standard) • Drug resistance testing possible • Differentiation of TB mycobacteria possible • Active TB detection	• Results take up to a month or longer • trained specialists, expensive infrastructure, uninterrupted power supply • Not applicable for LTBI • Less sensitive in children	[2,51,61,62]
Lateral flow immunochromatographic assay (TB-LAM)	• Rapid and user-friendly • POC-testing • High efficacy HIV patients • Active TB-detection	• Limited sensitivity & specificity • Reduced efficacy in HIV negative patients • Not applicable for LTBI • Less sensitive in children	[63]

**Table 2:** All included studies with their respective reference and index tests

Index-Test	Reference-Test						
	SSM	Culture	Xpert	CXR	IGRA	TST	No diagnostic
SSM	[24,25], [20], [21], [22] [*n* = 5]	[10], [20], [21] [*n* = 3]		[24] [*n* = 1]	[21] [*n* = 1]	[21] [*n* = 1]	[26], [27], [28] [*n* = 3]
Culture	[24], [20] [*n* = 2]	[20] [*n* = 1]		[24] [*n* = 1]			[26], [28] [*n* = 2]
Xpert	[11], [17], [24], [29], [18], [19], [30], [20], [12], [13], [21], [22], [15] [*n* = 13]	[10], [30], [20], [21] [*n* = 4]	[31] [*n* = 1]	[24], [29] [*n* = 2]	[21] [*n* = 1]		[27], [32], [28] [*n* = 3]
CXR	[24] [*n* = 1]		[33] [*n* = 1]	[24], [33] [*n* = 2]		[21] [*n* = 1]	[26], [34] [*n* = 2]
QFT/ IGRA	[16] [*n* = 1]	[16] [*n* = 1]		[16] [*n* = 1]	[16], [35], [36] [*n* = 3]		[26], [34] [*n* = 2]
TST	[16] [*n* = 1]	[16] [*n* = 1]		[16] [*n* = 1]	[35], [36] [*n* = 2]		[34] [*n* = 1]
TB-LAM	[14] [*n* = 1]		[31] [*n* = 1]			[21] [*n* = 1]	
TB-LAMP	[21] [*n* = 1]	[21] [*n* = 1]			[21] [*n* = 1]		
No dia-gnostic	[16] [*n* = 1]	[16] [*n* = 1]			[16] [*n* = 1]		

SSM = Sputum Smear Microscopy; Xpert = GeneXpert; CXR = Chest X-Ray; TB-LAM = Lateral flow immunochromatographic assay, TST = Tuberculin Skin Test, IGRA = Interferon-Gamma-Release Assay, QFT = QuantiFERON-TB, TB-LAMP = Loop-mediated isothermal amplification

### Test accuracy

Only five studies explicitly mentioned culture as the reference for test accuracy [10, 13, 15, 21, 28]. Only two studies used primary data from their trials, both originated from the United States and compared three-times sputum smear microscopy (3TSSM) with GeneXpert, both using culture as a reference from an institutional perspective in a hospital setting [10, 15]. Hickey et al. [15] reported a sensitivity of 69.4%, while Cowan et al. [10] reported 80% for 3TSSM. The sensitivity difference for GeneXpert was smaller but still notable, with 90.5% reported by Hickey et al. compared to 85% by Cowan et al. Conversely, the specificity differences between the studies and tests were minimal, ranging from 96.8 to 97% for 3TSSM and 98–100% for GeneXpert.

Two studies investigated Southern African populations with a high prevalence of HIV [13, 28]. Based on test accuracy data from previously published studies in the region [37, 38], the sensitivity of 3TSSM varied significantly between the two studies, at 50% and 28%, respectively. Differences in GeneXpert sensitivity were also observed, though less pronounced, at 83% and 73.3%. The specificity showed less variation, ranging from 96 to 100% for 3TSSM and 95–99.2% for GeneXpert. The study from Thailand by Chitpim et al. [21] used published WHO data for their analyses.

### Utility parameters

QALYs were chosen as the utility parameter in 7 out of the 17 cost-utility analyses [20–22, 26, 34, 36, 39]. All studies reported their utility weights along with the sources from which they were obtained, but only three studies used QALYs solely from sources that had derived them for the respective countries, as shown in Table 3. Differences in utility loss definitions were observed for several TB treatment states. Untreated active TB was weighted at -0.19 QALYs by Gosce et al., compared to -0.3 QALYs by Choi et al. and Li et al. [20, 26, 36]. When comparing weights for treated active TB, the differences were more pronounced, with values ranging from 0.69 to 0.85 QALYs [20, 21, 26, 36, 39]. Similarly, for cured TB and treatment-completed states, QALYs ranged from 0.80 to 0.94 [21, 36, 39]. Overall, we observed differences in QALY weights for each health state ranging from 0.11 to 0.16.

**Table 3:** QALY utility weights used in TB health economic evaluations

Study	Health state	Weight	Source
Choi et al. [20] US	TB-DST treatment (Medication regimen) TB-MDR treatment (Medication regimen) Treated active TB (6-Month-DST; 18-M-MDR) Untreated active TB Drug hepatotoxicity	0.9 0.7 0.85 0.7 0.8	Perio et al. 2009 US
Kowada et al. [34] JP	Dialysis with LTBI Dialysis with LTBI therapy Dialysis with LTBI therapy with complications Dialysis with non-fatal active TB before and during therapy	0.57 0.55 0.54 0.48	Tsevat et al. 1988 US Laupacis et al. 1996 CA
You et al. [22] CN	Active TB survival age 18–64 years Active TB survival age 65–85 years	0.92 0.84	Gold et al. 1998 US
Li et al. [26] HK	Treated active TB Untreated active TB Drug hepatotoxicity	0.85 0.7 0.8	Choi et al. 2013 US Guo et al. 2008 CA
Chitpim et al. [21] TH	EQ-5D: On TB treatment On MDR-TB treatment TB or MDR-TB cured or completed treatment TB with HIV, on TB treatment TB or MDR-TB with HIV cured or completed treatment EQ-VAS: On TB treatment On MDR-TB treatment TB or MDR-TB cured or completed treatment TB with HIV, on TB treatment TB or MDR-TB with HIV cured / treated	0.69 0.51 0.88 0.67 1.00 0.80 0.60 0.85 0.70 0.80	Kittikraisak et al. 2012 TH
Gosce et al. [36] BR, ZA, UK	Utility without TB (normal health) Utility loss due to untreated active TB Utility loss due to inpatient treatment Utility loss due to outpatient treatment Utility loss due to active TB treatment adverse effects Utility loss due to TBI treatment	0.88 -0.19 -0.210 -0.067 -0.17 -0.2	Mugwagwa T. et al. 2021 UK
Liu et al. [39] CN	LTBI Active TB Active TB cured / self-healed	0.97 0.82 0.94	Zu X. et al. 2020 CN

TB-DST = Tuberculosis drug susceptible treatment, TB-MDR = Tuberculosis multidrug resistance

DALYs were the primary utility parameter in eleven of the 17 cost-utility analyses. However, only seven studies reported their weights [11, 14, 18, 19, 30, 32, 33]. A detailed list of all DALY weights is presented in Table 3. The sources for DALY weights primarily included the WHO Global Tuberculosis Reports [1], the Global Burden of Disease (GBD) studies [30], and the 2021 study by Menzies et al. [40]. While DALY weights were similar across studies, the definition of effect parameters from which DALYs were derived substantially impacted the disability outcomes. For example, comparing the studies by Htet et al. [33] and Brümmer et al. [32], both analyzing TB cases averted by GeneXpert as an intermediate measure to assess DALYs, showed that Brümmer et al. considered only true positive cases, while Htet et al. included false positive and true positive cases in their assessment. Table 4 gives an overview of the disability weights assigned to different clinical and test outcomes in the respective studies.

**Table 4:** DALY disability weights used in TB health economic evaluations

Author	Health state	Weight	Source
Menzies et al. [30]	Active TB	0.271	WHO-GBD (2008), Murray et al. (1996)
Di Sun et al. [14]	TB TB therapy	0.264 0.1	WHO-GBD report (2008)
Shah et al. (18)	TB with HIV infection TB therapy	0.399 0.1 (0.2)	Salomon et al. (2012), Vassall et al. (2011), Di Sun et al. (2013)
Adelman et al. [11]	HIV with untreated TB HIV with treated TB HIV with MDR-treatment	0.399 0.1 0.2	Salomon et al. (2012), Shah et al. (2013)
Orlando et al. [19]	HIV + TB-patients	0.399	Shah et al. (2013)
Htet et al. [33]	DALYs averted per new active TB case detected. True positives + false positives	2.39	Menzies et al. (2021)
Brümmer et al. [32]	DALYs averted per true positive case	1.9	Azman et al. (2014), Menzies et al. (2021)

### Effect parameters

All effect parameters assessed in the included studies are presented with detailed definitions in Table 5. When examining years of life saved (YLS), we encountered three definitions [17, 28, 31]. Reddy et al. [31] assessed it based on the difference in life expectancies of compared groups. Lee and colleagues [17] defined it as life expectancy from the moment the individual enters the model until their death, while Andrews et al. [28] used monthly mortality rates based on a previously published cohort study. Overall, 15 of the 28 studies used effect parameters, of which nine used some form of TB detection based on test accuracy, but only six provided detailed definitions. Three studies defined correct TB diagnosis as true positives (TP) and true negatives (TN) [10, 15, 23], the other three restricted their analysis to true positive test outcomes, thus focusing only on patients correctly diagnosed with TB [11, 13, 32].

**Table 5:** Effect parameters used in TB health economic evaluations and how they were defined

	Effect parameter	Definition
Abimbola et al. [24]	Death averted during HIV treatment	Difference between survival rates in the base case and comparator
Andrews et al. [28]	Life years saved (YLS)	Life-expectancy based on “Monthly mortality probabilities”. Appendix indicates that the probabilities were taken from a prospective study in peri-urban Cape Town.
Cowan et al. [10]	Correct detection of PTB, Airborne infection isolation	True positives and true negatives (TP + TN) Duration of isolated hospital stay until release
Haukaas et al. [16]	Avoided PTB cases	Difference in number of TB cases under strategy 1 compared to number of TB cases under strategies 2, 3, or 4, but TB cases were not clearly defined.
Wikman-Jorgensen et al. [29]	Years of life lost (YLL)	YLL calculated through standard expected years of life lost approach with 82 years as the maximum life expectancy.
Adelman et al. [11]	TP, FN, FP	Additional TP diagnoses detected, averted FN and FP diagnoses.
Sohn et al. [35]	Number of active TB cases averted	Averted total TB cases through (a) LTBI screening or (b) preventive isoniazid therapy, compared to baseline scenario (no screening or therapy). Active TB cases were projected as LTBI prevalence times probability of progression from LTBI to active TB.
Lee et al. [17]	Years of life saved (YLS)	Model-based simulation of remaining live expectancy under each strategy.
Pooran et al. [13]	Number of cases diagnosed, initiated on treatment, and completing treatment	Based on number of culture positive TB cases: (1) Diagnosed by index, (2) Anti TB treatment, (3) TB treatment same day as diagnosis, (4) completed treatment, (5) having improved morbidity (Based on TB score).
Reddy et al. [31]	Years of life saved (YLS)	Difference in life expectancy under different strategies (not explicitly stated).
Hickey et al. [15]	True case detecting probability, TP and TN separately	TP, TN, true cases defined as TP or TN.
Htet et al. [33]	New active TB cases detected	Accuracy parameters to detect active TB with different diagnostic strategies based on National TB Prevalence Survey. Details are not given in the study, but cited literature indicated bacteriologically confirmed TB as reference (definitions were somewhat unclear as this may refer to a variety of diagnostics such as SM, culture or GeneXpert).
Brümmer et al. [32]	Case detection	TP case detection No averted DALYs for FN, and no averted DALYs but treatment cost for FP treatment.
Navarro et al. [23]	Correctly diagnosed cases	Correctly diagnosed cases include the group of true-positive and true-negative subjects.
Liu et al. [39]	a) Misdiagnosis rate b) Omission diagnostic rate c) The number of patients correctly classified d) The number of tuberculosis cases avoided	(a) Misdiagnosis rate: Proportion of healthy individuals diagnosed as TB cases in participants; (b) Omission diagnostic rate: Proportion of undiagnosed TB cases (LTBI and active TB) in participants; (c) The number of patients correctly classified: Number of correctly diagnosed positive TB cases; (d) The number of tuberculosis cases avoided: Projected number of active and latent TB cases expected to progress to active TB, correctly diagnosed and treated, thus expected not to further spread the disease.

### Cost parameters

Only three of the studies included in this review explicitly mentioned their cost valuation method [12, 13, 35]. Pooran et al. [13] used a solely bottom-up approach, whereas Vassall et al. [12] and Sohn and colleagues [35] employed a combined top-down and bottom-up methodology. Eight studies specified their approach for cost measurement, all employing micro-costing methods [12, 13, 17, 27–30, 35].

Based on these dimensions of cost valuation methods, we adjusted the 2 × 2 table created by Tan et al. to illustrate the strengths and weaknesses of each valuation option in Table 6. Any study in this review mentioning their cost valuation method is cited respectively [41].

**Table 6:** Strengths and weaknesses of each cost valuation method

	Resource use – Accuracy +	
**Unit cost** **+** Accuracy **–**	**Top-down Gross Costing** Pros: • Simple, fast, minimal data necessary • Good for high-level budgets Cons: • Low accuracy • Lacks granularity, hard to find inefficiencies Studies: None	**Top-down Micro-costing** Pros: • More detailed than gross costing • Moderately resource intensive Cons: • Still generalized, less accurate • Requires more effort in the data collection process Studies: (12,13,17,27–30,35)
	**Bottom-up Gross Costing** Pros: • More accurate, patient/service level focus • Identifies broad inefficiencies Cons: • Needs more data than top-down • May miss detailed cost variations Studies: None	**Bottom-up Micro-costing** Pros: • Most detailed costing method • Capturing individual variations & identify true cost drivers Cons: • Highly resource intensive • Complex data collection, not practical for variable overheads like catering, laundry, supervision Studies: (12,13,17,27–30,35)

While all studies presented the data sources for their cost positions, the level of detail varied. In 17 of the 28 studies, the authors included a dedicated section on how cost data was acquired [12, 13, 17, 18, 20, 21, 23, 25–31, 33, 35, 36]. The remaining studies, however, only reported cost data in tables, referring to the sources as “from published literature”. While this approach is understandable, it may pose challenges for readers to fully grasp the method, form, or setting in which this information was obtained without a thorough review of the referenced sources.

## Discussion

This narrative review explored the methodological and diagnostic approaches used in health economic evaluations of TB diagnostics. An important observation from this review is that only 10 of the 28 included studies explicitly state the type of TB. This information can, to some extent, often be inferred indirectly from other details, such as the selection of diagnostic reference and index tests. However, TB is a highly complex disease that can affect multiple organs and become active after being inactive in infected but asymptomatic individuals for a very long period. To bridge this gap, future studies may explicitly state the type of TB being examined, whether it is active or latent, PTB or EPTB with its respective forms (e.g. lymph node TB, pleural TB, skeletal TB, genitourinary TB). This could be achieved by including a standard TB disease classification section in health economic evaluations, ensuring clarity regarding which form of TB is targeted by the diagnostic tests.

The majority of included studies were model-based evaluations. Essential model parameters for the simulation of cohorts, such as disease type, prevalence, and test accuracy, were taken from published literature. While this is an adequate and practical approach, model parameters must be selected carefully to obtain unbiased and precise predictions for the simulated scenarios. Due to the complex nature of TB, the target population should be explicitly stated, as screening and diagnostic strategies vary significantly among different groups. Especially in detecting LTBI, host genetic factors can alter test sensitivity [42].

Hailu et al. highlighted another factor affecting test accuracy. They found significant variations in TB prevalence rates among seemingly similar populations [43], which can substantially influence the accuracy of diagnostic tests [44]. Additionally, differences in patient characteristics and underlying settings, as well as potential measurement errors, need to be taken into account when making assumptions about the test accuracy [44]. To enhance the reliability of model-based evaluations, future studies should ensure that model variables such as disease prevalence, disease severity, patient demographics, and test accuracy are derived from well-defined, contextually relevant data sources.

For test accuracy measurements, only five of the included studies utilized culture, the current gold standard, as their reference test. Among these, variations in test accuracy were observed, even in studies conducted in seemingly similar settings, such as the two studies conducted in U.S. hospital care settings by Hickey et al. [15] and Cowan et al. [10], or the South African studies by Pooran et al. [13] and Andrews et al. [28]. A careful evaluation of the sources for assumed parameters and a transparent description of their potential limitations, together with extensive sensitivity analyses, are essential for comparable and interpretable information.

Effect parameters differed in their definitions from study to study, which limited their comparability and transferability across settings. All studies using QALYs reported the underlying utility weights, whereas only seven out of 17 studies using DALYs did so. QALY weights varied slightly between studies, likely due to regional preferences influencing these weights. Some studies derived DALYs from intermediary effect parameter outcomes, which can vary significantly and impact the outcome. In order to improve consistency and comparability across studies and enhance the transferability of model parameters for secondary data analyses, future research should pursue a standardization of definitions for effect parameters. This includes consistently reporting the utility and disability weights and intermediary outcome measures.

The choice of methods for the valuation and measurement of costs is crucial for ensuring the accuracy, i.e., internal and external validity [9] and the statistical precision of cost assessments. For example, a top-down valuation means estimating average costs for full sets of products and services, which is considered less accurate. However, it may achieve high statistical precision, as centralized accounting departments may include large numbers of invoices in the estimation of average costs. Conversely, a bottom-up approach measures costs through direct observation of patients and processes highly detailed cost data. It is, however, resource-intensive and limited to what is observable to the field team among the patients included in the respective study. Furthermore, bottom-up approaches bear the risk of missing, for example, overhead costs like catering or laundry services. Combinations of bottom-up and top-down approaches may mitigate the potential shortcomings of either of the approaches [9] and avoid the risk of underestimating the true costs of a test or an intervention [45]. Despite this, the standardized cost terminology from CHEERS was used only in a fraction of the reviewed studies. Only three studies mentioned the valuation methodology [12, 13, 35], and only eight studies stated the measurement approach [12, 13, 17, 27–30, 35].

A further challenge arises from the use of secondary cost data without providing sufficiently detailed descriptions of how data were obtained in the referenced literature. This makes assessing the accuracy and precision of the adopted cost data difficult without extensive additional reading. For instance, one study published in 2022 employed cost data from a study already published in 2013 [15, 20]. While adopting cost data from the literature is agreed to be reasonable and convenient, methods for assessing cost inflation, changes in healthcare policies, technological advancements, including new medical treatments and diagnostic options, and adjustments in reimbursement and subsidy rates may change cost structures and jeopardize transferability across different settings. For example, subsidies from external organizations may bias the observed costs downwards, thereby altering the cost-effectiveness in favor of the evaluated technology. While this is in itself unproblematic, the transportation of those cost data into settings when a test is not subsidized may produce biased results and lead to an underestimation of the true cost [46]. The comparability of evaluation studies is further hindered by significant variability in discount rates for utility and cost parameters. In the studies included in this review, discount rates ranged from 0 to 5%, with some studies discounting only either costs or utilities, while others did not specify whether they discounted at all. These factors should be critically appraised when adopting cost data, and market prices should be used in sensitivity analyses to give an upper-cost limit as a conservative estimate.

WTP thresholds are often used to allow statements about whether or not adopting a new test or test strategy would be reasonable, given the estimated costs per QALY or DALY in an evaluation. In addition to the potential impact of different cost measurement and discounting methods, WTP thresholds can be obtained through different approaches with different results. For example, stated and revealed preferences may differ considerably, and both may deviate drastically from the WHO threshold based on a country’s per-capita GDP (gross domestic product). The source and implications of WTP thresholds should therefore be carefully considered, and recommendations should be communicated carefully to policymakers, with clear explanations of how and why the respective thresholds were chosen. Both researchers and policymakers also need to be aware that the assessments of whether or not technologies or strategies are cost-effective, as well as the decisions whether or not they should be implemented, are inherently normative in policymaking, and that WTP thresholds can only serve as a point of reference for these decisions.

A couple of challenges were addressed by virtually none of the included studies. The first challenge is related to sequential testing, where the test under consideration may be one piece in a predetermined series of successive tests. Since test accuracy depends on the disease prevalence in the tested population [44], the specificity and sensitivity of each test in the series depend on the position in the sequence and the accuracy of the preceding tests. This can be particularly challenging with a complex disease like TB, where sequential and composite testing strategies are common to detect this difficultly diagnosable disease. This also needs to be considered in trials evaluating new tests, when a positive outcome from what can be called a gatekeeper test is among the patient inclusion criteria. Neglecting this can lead to biased results and misleading interpretations [47].

The second challenge was, in a broader sense, the cost of inaction regarding TB, which can occur in cases where an infected patient is not diagnosed by a positive test result. We are aware of one recent study from India, which comprehensively assessed the cost of missing and thus not treating TB-positive cases [48]. From a societal perspective, taking all potential costs, including productivity losses and losses in quality of life for additionally infected individuals, one missed TB case was estimated to cost at least three to four times what TB treatment for one patient would cost. The methodologically pioneering and feasible assessment of the broader societal costs of missed TB cases, together with the considerable economic impact found, may encourage researchers to include this overlooked issue in future studies [48].

Similarly, an inconsistent consideration of costs associated with FP results was observed, where further unnecessary diagnostic procedures and the initiation of treatments were not taken into account. Lastly, our selected studies did not account for process utility prior to testing. The disutility stemming from uncertainty about one’s future health before and during the diagnostic procedure can range from 0.0005 to 0.031 QALYs [49]. However, QALYs may be inadequate in fully capturing the accompanying anxiety experienced prior to testing, as their weight assessment methods are based on stable health states over time. This can result in an underestimate of the true burden experienced by individuals before test results are known [50]. 

The strength of this review lies in its comprehensive extraction of key study characteristics for each health economic evaluation, particularly focusing on the effect and cost parameters, their definitions, data extraction methods, and assessments. Nonetheless, this review has several limitations. The studies included in this review were retrieved using various search terms and strategies without a systematic approach, providing only a snapshot of health economic evaluations in the field of TB. This may have introduced some bias in the selection process, which may have favored more easily accessible or more prominently indexed studies. This may limit the comprehensiveness of our findings and could make replications or updates of this work challenging in the future. While this would be problematic in systematic reviews, where the overall outcome, such as a broader picture of the cost and utility of a technology, remains the focus, we consider it unproblematic here because the aim is to highlight the different uses of similar terms and parameters across well-published studies in the field.

## Conclusion

Precise terminology and clear definitions of parameters and methodologies in health economic evaluations are necessary, especially in the field of TB diagnostics, to generate evidence to guide policymakers and support clinical decision-making. Transparency and clarity about differences in populations, study settings, selection of reference tests, definitions of effect parameters, and cost valuation methods, are essential to ensure the comparability of results from different decades, continents, healthcare systems, and technologies. The discussed studies exhibited a certain degree of variation in how detailed the information was presented. While the overall quality of the included studies was as solid as can be expected in peer-reviewed literature, missing out on some of the details and assumptions may limit the usefulness of some studies unless readers undertake further efforts to fill potential blanks. Future researchers and decision-makers should consider the impact of sequential diagnostics on test accuracy, as well as the cost of inaction and FP cases in their endeavor to eliminate TB.

## Electronic supplementary material

Below is the link to the electronic supplementary material.

Supplementary Material 1

## Data Availability

No datasets were generated or analysed during the current study.

## Abbreviations

CBA: Cost benefit analyses
CEA: Cost effectiveness analyses
CHEERS: Consolidated health economic evaluation reporting standards
CT: Computed tomography
CUA: Cost-utility analyses
CXR: Chest X-ray
DALY: Disability-adjusted life year
EPTB: Extrapulmonary tuberculosis
FN: False negative
FP: False positive
GBD: Global burden of disease
GDP: Gross domestic product
HIV: Human immunodeficiency virus
ICER: Incremental cost-effectiveness ratio
IGRA: Interferon-gamma release assay
LTBI: Latent tuberculous infection
MTB: Mycobacterium tuberculosis bacterium
NAAT: Nucleic acid amplification test
PCR: Polymerase chain reaction
PTB: Pulmonary tuberculosis
QALY: Quality-adjusted life year
SSM: Sputum smear microscopy
3TSSM: Three times sputum smear microscopy
TB: Tuberculosis
TB-DST: Drug susceptible tuberculosis treatment
TB-MDR: Multidrug-resistant tuberculosis treatment
TN: True negative
TP: True positive
TST: Tuberculin skin test
WHO: World health organization
WTP: Willingness-to-Pay
YLS: Years of life saved
YLL: Years of life lost
