# Supplementary material for: Health economic evaluations of diagnostic tests for tuberculosis: a narrative review
Source: Health Econ Rev. 2025 May 24;15:43. doi: 10.1186/s13561-025-00639-2 (PMC12102823; doi:10.1186/s13561-025-00639-2)
Supplement: Supplementary file 1 — Supplementary Material 1 [file 13561_2025_639_MOESM1_ESM.docx]

**Supplement to
Challenges in Health Economic Evaluations of Diagnostic Tests for Tuberculosis: A Narrative Review**

Cemre Arpa^1,2^, Ahmed Abd El Wahed^3^, Serap Aydin^1^, Prakash Ghosh^1,3^, Dinesh Mondal^4^, Lydia Nakiyingi^5^, Julius Boniface Okuni^6^, Sophie Schneitler^7^, Manfred Weidmann^8^, Martin Siegel^1,2^

*^1^Department of Empirical Health Economics, Technische Universität Berlin, Berlin, Germany*

*^2^Heidelberg Institute of Global Health, Heidelberg University, Heidelberg, Germany*

*^3^Institute for Animal Hygiene and Veterinary Public Health, Leipzig University, Leipzig, Germany, ^4^International Centre for Diarrheal Disease Research Bangladesh (icddr,b), Dhaka, Bangladesh*

*^5^Makerere University College of Health Sciences*

*^6^College of Veterinary Medicine, Animal Resources and Biosecurity, Makerere University, Kampala, Uganda.*

*^7^Institute of Medical Microbiology and Hygiene, University Clinic Saarland, Homburg (Saar), Germany,*

*^8^Midge Medical GmbH, Berlin, Germany*

*Corresponding Author: Martin Siegel, E-Mail:* [*martin.siegel@tu-berlin.de*](mailto:martin.siegel@tu-berlin.de)

Table S 1: Detailed study characteristics of included review articles

| Author | Population | | | | TB type | Model | | Perspective | | Discount rate | | Time horizon in years |
| --- | --- | --- | --- | --- | --- | --- | --- | --- | --- | --- | --- | --- |
| Abimbola et al. (36) | Sub-Sahara Africa. Early detection of active TB during ART initiation and reduce early mortality among PLHIV who present to care with advanced HIV. | | | | Active TB detection during HIV treatment process | CEA – Decision tree | | Health system | | 3% (utilities only) | | 0.33 |
| Reddy et al. (43) | Malawi, South-Africa - Hospital setting, HIV positive patients (Simulated cohorts of unselected hospitalized patients with HIV) | | | | Active TB | CEA –  The Cost-Effectiveness of Preventing AIDS Complications (CEPAC) [Micro-simulation model] | | NA | | 0%; 3%  (both tested) | | 2; 5; lifetime |
| Cowan et al. (9) | USA – Hospital setting, 318 AII (airborne infection isolation) inpatients | | | | PTB | CEA – Decision tree | | Institutional perspective | | NA | | Release from AII |
| Haukaas et al. (15) | Norwegian - Immigrants <35 from high incidence countries 40/100,000 per year | | | | LTBI & PTB | First through Decision tree than Markov model, CEA | | Healthcare perspective | | 4% - only cost | | 10 |
| Author | | Reference strategy | Index strategy | Cost parameters | | | Utility parameters | | WP-Threshold | | ICER | |
| Abimbola et al. (36) | | 1.Clinical diagnosis (symptoms screening) + Smear Microscopy + CXR | 2.Smear-Micoscopy + CXR + Culture 3. Xpert MTB/RIF | Valuation method (VM): NA  Measurement (M): NA  Data source (DS): Published literature | | | Death averted: Difference between survival rates in the base case and comparator | | South Africa GDP/person  US$ 5678 | | Strategy 2: Averted 2 more death, but more costly  Strategy 3: Averted 1 more death and cheaper. | |
| Reddy et al. (43) | | Xpert | Sputum-Xpert MTB/RIF + Urin-TB-LAM + concentrated.  Urin-Xpert | VM: Bottom-up (Implicit)  M: Micro costing (Implicit)  DS: STAMP-Study, country-specific costing studies and national laboratory listings | | | Years of life safed (YLS) | | US$750/year of life saved (YLS) in Malawi and $940/YLS in South Africa | | $450 per YLS in Malawi and $840 per YLS in  South Africa | |
| Cowan et al. (9) | | Culture | 1. 1x Xpert MTB/RIF with unconcentrated Sputum 2.1x Xpert with concentrated Sputum  3. 2x Xpert with concentrated Sputum 4. 2x Smear Microscopy 5. 3x Smear Microscopy | VM: NA  M:NA  D: AII duration from own study, otherwise published literature  Penalty for FN results included | | | Right detection of TB (diagnostic accuracy – TP & TN) and duration until hospital release. | | 50,000$ | | Xpert on 1 unconcentrated sputum sample most cost-effective; $11466 relative to microscopy. | |
| Haukaas et al. (15) | | (IGRA) and CXR, if + --> Culture & SM | 1. No screening LTBI  2. TST & IGRA  3. IGRA  4. IGRA risk population  [In all cases CRX] | VM: NA  M: NA  DS: Salary for employees in the health trusts, published literature, DRGs, expert opinion | | | Avoided TB cases:  N of TB cases that occur in the strategy 1 minus N of TB cases in strategy 2,3 or 4. | | NA | | TST + IGRA = ED IGRA risk = 3043€ IGRA = 28,303€ | |

| Author | Population | TB type | Model | Perspective | Discount rate | Time horizon in years |
| --- | --- | --- | --- | --- | --- | --- |
| Jun Li et al. (38) | A hypothetical cohort of 65-year-old people. (Hong Kong) | LTBI & active TB | Decision tree and Markov model  CEA + CUA | Health service provider | 5% | 20 |
| Sohn et al. (47) | Korean population of 10,000 after TB outbreak. (13–18 year) | LTBI & active TB | CEA – Decision tree | Health system | 3% (only cost) | 2 |
| Lee et al. (16) | Simulated Indian adult cohort. Aged ≥15 years with cough of ≥2 weeks duration. | PTB | The Cost-Effectiveness of Preventing AIDS Complications (CEPAC)  [Microsimulation model] | Health system | 0 and 3% | 5 |
| Adelman et al. (10) | Ethiopian HIV-Clinic setting with 828 HIV+ patients | LTBI & active TB | CUA – Decision tree | NA | 3% only utilities | 0.33 |

| Author | Reference strategy | Index strategy | Cost parameters | Utility parameters | WP-Threshold | ICER |
| --- | --- | --- | --- | --- | --- | --- |
| Jun Li et al. (38) | No Screening | 1. CXR + smear & culture (TB-screening)  2. Xpert MTB/RIF (TB-Screening) 3. Consultation + CRX & Smear& Culture (No screening) 4- QuantiFERON®-TB Gold In-Tube (QFT-GIT) + CXR (LTBI-screening) | VM: NA  M: NA  DS: Hospital Authority charges, published lit., gov. and annual census | Years of life saved (YLS) & QALYs, weights: treated active TB - 0.85  Untreated active TB - 0.7, drug  hepatoxicity - 0.8 | US$50,000 per QALY gained | LTBI /TB screening - 29.951/QALY |
| Sohn et al. (47) | IGRA | 1) TST alone  2) Stepwise TST/IGRA for TST-positive (.10mm) individuals.  Gold standard=QFT-GIT  [Accuracy based on published literature] | VM: Top-down and bottom-up (Mixed-method)  M: Micro-costing (Ingredient-based)  DS: Consultation, guidelines, literature | Total N of TB cases averted: No screening vs different strategies  Test independency assumed. Assume: No intervention results in 4.4 TB cases / 1000 | NA | IGRA: US$140 933/TB case  TST/QFT-GIT: Dominated  TST: Most effective |
| Lee et al. (16) | (1) Sputum smear microscopy (SM) in microscopy centers (DMCs) | (2) Xpert MTB/RIF in DMCs (Xpert); (3) Truenat in DMCs (Truenat DMC); and (4) Truenat for point-of-care testing in primary healthcare facilities (Truenat POC). | VM: NA  M: Micro-costing  DS: Guidelines, published literature | YLS: remaining live expectancy from when an individual enters the model until his death, under each strategy | ICER was <US$990/YLS | SSM dominated Xpert & Truenat in DMC; Truenat POC most cost-effective. |
| Adelman et al. (10) | 1. Symptom screening + smear microscopy + clinical diagnosis | 2.Symptom screening + Xpert MTB/RIF  [Accuracy based on published literature, different populations] | VM: NA  M: NA  DS: Hospital, research institute, ministry of health | DALYs, additional TP diagnoses and FN and FP diag. averted,  HIV+& untreated TB-0.399, HIV+& DST TB-0.1, HIV+ & MDT-0.2 | 505$ per person (Based on GDP of Ethiopia) | Index strategy identified more TB cases and is extremely efficient with 5$/DALY |

| Author | Population | TB type | Model | Perspective | Discount rate | Time horizon in years |
| --- | --- | --- | --- | --- | --- | --- |
| Wikman-Jorgensen et al. (41) | Rural area in Mozambique. 5% HIV prevalence. | TB | CUA – Markov model | Health system | 3 | 90 |
| Shah et al. (17) | Simulation 10,000 Ugandans with HIV + and TB symptoms. | EPTB, PTB, disseminated TB | CUA – Decision tree | Health system | 3 | 1 |
| Kelly et al. (37) | Indian setting assumed prevalence 20%. | Active TB | CUA – Decision tree | Health system | 0 | 1 |
| Orlando et al. (18) | Simulation of 1000 HIV+ patients in Mozambique. | PTB | CUA – Decision tree | Health system | 3 | 1 |
| Zwerling et al. (39) | Malawian population with HIV+, prevalence 1%, 2.4%(cohort) and 6% scenario looked at. | Active TB | CUA– Decision tree | Health system | 3 | lifetime |

| Author | Reference strategy | Index strategy | Cost parameters | Utility parameters | WP-Threshold | ICER |
| --- | --- | --- | --- | --- | --- | --- |
| Wikman-Jorgensen et al. (41) | 1. 2x smear microscopy (SM)+ CXR | 2. Microscopic observation drug-susceptibility (MODS)  3. Standard care + confirmatory Xpert | VM: NA  M: Micro-costing  DS: Invoices from local and international providers, consultation,  ministry of health, published literature | YLL (standard expected years of life lost approach, 82 years) &DALYs | Mozambique gross national income pp.:  589$ (2013) | MODS 5374.58 $/ DALY averted.  Xpert:  122.13 $ / DALY |
| Shah et al. (17) | 1. 2x SM (Ziehl-Neelsen (ZN)) | 2. SM + LF-LAM  3. Xpert MTB/RIF  4. Xpert+ LF-LAM | VM: NA  M: NA  DS: Parent study, direct observation, invoices | DALYs averted, weights:  TB+HIV infection- 0.399  TB-treatment– 0.1 (0.2) | GDP pp.:  487$ | 2.: 33$/DALY  3.: 58$/DALY  4.: 57$DALY |
| Kelly et al. (37) | 1. SM-ZN | 2. SM-LED (light emitting diode  technology-based fluorescence microscopy (LED) | VM: NA  M: NA  DS: Testing center, own study and published literature | DALYs averted  Active TB 16.43  Treated TB 13.18 | GDP pp.:  US$ 1489  (2012) | 2.: US$14.64/DALY averted |
| Orlando et al. (18) | 1.Symptom screening + SM | 2. Xpert MTB/RIF  3. LF-LAM or Xpert based on N of CD4 cells | VM: NA  M: NA  DS: DREAM program,  published literature, Global Fund for TB | DALYs, weights:  TB – 0.399  AIDS care – 0.053 | GDP pp.:  382 $ (2016) | 2.: $56.54/DALY  3.: $72.31/DALY |
| Zwerling et al. (39) | 1. No standard care given, assumption: Clinicians assessment 66% probability of future treatment | 2. Xpert MTB/RIF  3. SM-LED | VM: NA  M: Micro-costing  DS: Parent study, budgetary reviews, interviews and logs of study staff, published literature, observations | DALYs, weights:  1^st^ line treatment success: -1.53; 2^nd^:-1.98  Death: -23.89  FP treatment: -1.27 | GDP pp.:  1417$ (2010) | N=50/100/1000  Prevalence 2.4%  2. $2809/ $1615/ $564  3. $1808/ $1216/ $699 |

| Author | Population | TB type | Model | Perspective | Discount rate | Time horizon in years |
| --- | --- | --- | --- | --- | --- | --- |
| Menzies et al. (42) | Simulated HIV+ cohort (Botswana, Lesotho, Namibia, South Africa, and Swaziland) | LTBI & active TB | CUA – Markov model | Health system | 3% | 10 and 20 |
| Choi et al. (19) | Simulated US cohort | PTB | CUA – Decision tree | Health system | 3% (only utilities) | 1 |
| Vassall et al. (11) | Using data from South African study with 4656 patients recruited for TB screening in primary health-care clinics (Of whom 62% were HIV+) | TB | CUA | Societal perspective | 0 and 3% | <1 |
| Pooran et al. (12) | 1502 participants form South Africa, Zambia, Zimbabwe, and Tanzania (TB-NEAT-trial) HIV+ patients included. | TB | CEA | Health system | 3% (only cost) | NA |

| Author | Reference strategy | Index strategy | Cost parameters | Utility parameters | WP-Threshold | ICER |
| --- | --- | --- | --- | --- | --- | --- |
| Menzies et al. (42) | 1. SM if + 🡪 treatment, if – in patients with TB history 🡪 culture test | 2. Xpert MTB/RIF | VM: NA  M: Micro-costing (Ingredient-based)  DS: WHO, literature, guidelines, nat. program | DALYs, weights:  Active TB – 0.271 | Ranging from 982$(Lesotho) - 7000$ (South Africa) GDP pp. | Over 10 years:  1257$ (B), 1011$(L), 878$(N), 958$ (SA),  792$ (S) / per DALY averted |
| Choi et al. (19) | 1. SM + clinical diagnosis (Cd) + confirmatory with culture for DST | 2. SM +MTD/Cd + culture  3. Strategy 2 without SM  4. SM + Xpert/Cd + culture 5. Strategy 4 without SM | VM: NA  M: NA  DS: Direct observation (Laboratory), literature, manufacturer/distributor, invoices & budget records (Out-patient) | QALYs, weights:  TB-DST- 0.9  TB- MDR– 0.7  Active TB – 0.85  Untreated act. TB- 0.7  Drug hepatotoxicity 0.8 | $50 000 per QALY | 1 - Dominated by all  2 – Used as reference  -vs 3= 47 914$/Q  -vs 4= 23 111$/Q  -vs 5= 39 992$/Q |
| Vassall et al. (11) | 1. SM | 2. Xpert MTB/RIF | VM: Bottom-up & Top-down  M: Micro-costing  DS: Primary participant, laboratory services, health facilities, published literature | DALY | Various thresholds scenarios | Xpert cost-neutral:  Xpert equipment and  tests were mitigated by a reduction in costs elsewhere in  the tuberculosis cascade of care. |
| Pooran et al. (12) | 1.SM | 2. Xpert  [Accuracy based of culture] | VM: Bottom-up  M: Micro-costing (Ingredient-based)  DS: Primary participant data | N of culture + TB cases: (1) diagnosed by index  (2) Anti TB treatment, (3) TB treatment same day as diagnosis, (4) completing treatment  (5) Having improved morbidity (Based on TB score) | Various thresholds scenarios | With 90% probability:  Xpert preferred if WP threshold higher than  (1) $9450/culture per + patient diag.,  (2) $4450 pp.,  (3) $1600 pp.  (4) $3820 pp.,  (5) $5840 pp., |

| Author | Population | TB type | Model | Perspective | Discount rate | Time horizon in years |
| --- | --- | --- | --- | --- | --- | --- |
| Di Sun et al. (13) | Inpatient hospitals in South Africa and Uganda with HIV+, prevalence 38% | Active TB | CUA | Health system | 3% | Lifetime |
| Kowada et al. (46) | Hypothetical cohort of Japanese hemodialysis patients age ≥40 years | LTBI & active TB | CUA – Decision tree + Markov model | Societal perspective | 3% | Lifetime |
| You et al. (21) | Hypothetical Chinese clinical cohort of suspected TB patients | Active PTB | CUA – Decision tree | Health system | 3% (cost only ) | 1 |
| Andrews et al. (40) | Hypothetical South African cohort initiating HIV+ treatment, prevalence 22%, | LTBI & active TB | CEA | NA | 3 | NA |

| Author | Reference strategy | Index strategy | Cost parameters | Utility parameters | WP-Threshold | ICER |
| --- | --- | --- | --- | --- | --- | --- |
| Di Sun et al. (13) | SM + Clinical diagnosis + further diag. tests | SM+ Clinical diagnosis + further diag. tests including LAM [all cases without culture] | VM: NA  M: NA  DS: Published literature | DALYs, weights:  TB- 0.264  TB treatment – 0.10 | GDP per person:  U: 509$  SA: 7275$ | Uganda: 208$/DALY  South Africa: 353$/DALY |
| Kowada et al. (46) | NA | 1. CXR  2. TST + CXR  3. QFT + CXR | VM:  M:  DS: Published sources, direct & indirect costs from ministry of health, published literature, Medical Insurance Reimbursement Table | QALYs, weights:  Dialysis with LTBI- 0.57,  Dialysis + LTBI without therapy complications – 0.55 and with complications – 0.54, dialysis with non-fatal active TB - 0.48 | Using US threshold for unknown reason 50,000/QALY | QFT strategy dominated both strategies |
| You et al. (21) | 1. 2x SM + Clinical diagnosis | 2. Either SM or Xpert positive (Both done)  3. Xpert | VM: NA  M: NA  DS: Hospital charges  [Hospitalization and intensive unit cost not included, Xpert cost estimated] | One-year mortality rate, life years gained (life expectancy in Hong Kong - age), QALYs 18-64 years: 0.92, 65-85 years:0.84 | Using US threshold for unknown reason 50,000/QALY | 1- Dominated by 2&3  2- Reference  3- 99$/QALY |
| Andrews et al. (40) | 1. No screening | 2. 2x SM symptomatic  3. 2x SM all patients  4. 2x Culture symptomatic  5. 2x Culture all  6. 1x Xpert symptomatic  7. 2x Xpert symptomatic  8. 1x Xpert all  9. 2x Xpert all | VM:  M: Micro-costing:  DS: Published literature (Unit costs), laboratory services | Sensitivity based on culture.  YLS-Years of life saved | South Africa’s GDP per person  7,100$/YLS | 2- 2,600$/YLS  3- 3,800$/YLS  5- 5,060$/YLS  9- 5,140$/YLS  Rest dominated |

| Author | Population | TB type | Model | Perspective | Discount rate | Time horizon in years |
| --- | --- | --- | --- | --- | --- | --- |
| Hickey et al. (14) | US - Hospital setting  Retrospective study  Eligible patients were adults (age ≥18 years) | PTB | CEA – Decision tree | Hospital | NA | NA |
| Htet et al. (45) | Malaysian population. Secondary data from the National TB Prevalence Survey of Myanmar (2010 and 2018) and data from a community-based cross-sectional TB screening survey. National TB prevalence of 468 per 100,000 | Active TB | CUA – Decision tree | NA | 3%  (cost only) | NA |
| Liu et al. (51) | Chinese population: A high-risk population of MTB infection (contact, HIV, immunosuppressive therapy), hypothetical cohort of 10,000 | LTBI & active TB | CEA & CUA | Societal perspective | Not applied | 1 |

| Author | Reference strategy | Index strategy | Cost parameters | Utility parameters | WP-Threshold | ICER |
| --- | --- | --- | --- | --- | --- | --- |
| Hickey et al. (14) | 1.SM 3x (Culture use for accuracy testing) | 2. Xpert 1x  3. Xpert 2x  (If any + then +) | VM: NA  M: NA  DS: Kaiser Family Foundation data for New York State hospitals, Choi et al. 2013 | Additional probability of detecting:  TP & TN (True case), TP and TN separately | NA | 2. Strategy  TC: 3.6 (2.5–4.7)  TP: 0.5 (−0.1 to 1.1)  TN: 3.1 (2.2–4.0  3. Strategy less cost-effective see tab.4 |
| Htet et al. (45) | TBSS screening (TB signs and symptoms) 1- If TB symptoms -> care seeking (yes/no)-> if yes CXR -> if yes Xpert->if yes treatment | 2. Mobile App + CXR  3. All CXR | VM: NA  M: NA  DS: Secondary data from the National TB Prevalence Survey of Myanmar (2010 and 2018) and data from a community-based cross-sectional TB screening survey. | New active TB cases detected, DALYs  New active TB cases detected by each screening strategy were  defined as individuals positive for M tuberculosis in the Gene Xpert MTB/RIF assay.  DALYs = 2.39 * new TB case detected | GDP per capita:  1x: 1477.50$  2x: 2955$  3x: $4432.50  Per DALY | Mobile app - 1064$/DALY  CXR - 3143$/DALY |
| Liu et al. (51) | 1. TST: Tuberculin pure protein derivative (TB-PPD) | 2. Recombinant Mycobacterium tuberculosis fusion protein (EC) | VM: NA  M: NA  DS: China Pharmaceutical Information Database (EC &TB-PPD), published literature | 1^st^: QALYs 2^nd^: Diagnostic effectiveness, misdiagnosis rate, the omission diagnostic rate, N of patients correctly classified, N of TB cases avoided. | <80,976 CNY per QALY,  Still acceptable if in range of 80,976- 242,928 CNY | Strategy 1 was dominated  EC-1^st^: 192,043.60 CNY per QALY  EC-2^nd^: 7,263.53 CNY per misdiagnosis rate reduction |

| Author | Population | TB type | Model | Perspective | Discount rate | Time horizon in years |
| --- | --- | --- | --- | --- | --- | --- |
| Navarro et al. (22) | Bacillus Calmette-Guérin (BCG) vaccinated population in Colombia - simulated --> Lowers TST specificity | LTBI & PTB | CEA – Decision tree | General system of social security in Health  Health system | Not applied | 0.5 |
| Gosce et al. (48) | Brazil, South Africa, UK - Simulated cohort 100,000 people without active TB (of all ages) get tested for TB infection. | Active TB | CUA- Markov model | NA | UK: 3.5%, Brazil: 5%,  South Africa: 3% | 20 |
| Chitpim et al. (20) | Thailand: General population imitated Cohort >15y with + CRX and Clinical observation | PTB | CUA-Decision tree and Markov model | Societal perspective | 3% | Lifetime |
| Brümmer et al. (44) | Simulated cohort of 100,000. For hypothetical country scenarios with respective prevalence (India, Philippines, South Africa, Uganda, Vietnam) | TB | CUA –  Markov model | Healthcare system | 3% | 10 |

| Author | Reference strategy | Index strategy | Cost parameters | Utility parameters | WP-Threshold | ICER |
| --- | --- | --- | --- | --- | --- | --- |
| Navarro et al. (22) | 1. TST + CXR | 2.QuantiFERON-TB Gold Plus (QFT-Plus) + CXR | VM: NA; M: NA  DS: Capitation Payment Unit database, Ministry of Health | Correctly diagnosed cases. (CDC) CDC included the group of true-positive and true-negative subjects. | GDP per capita (2022):  $6666USD | Per additionally correct diagnoses per strategy 2 the system would need to pay $5687. |
| Gosce et al. (48) | 1. Diaskin-test (DST) | 2. TST  3. IGRA QFT | VM: NA  M: NA  DS: Published literature, manufacturer, NHS tariffs (TBI treatment costs), Steffen et al. 2020 (Brazil) | QALYs, weights:  Without TB (healthy) 0.88  Untreated active TB -0.19  Inpatient treatment -0.210  Outpatient treatment-0.067  Active TB treatment adverse effects 0.17  Utility loss due to TBI (Infection) treatment 0.2 | Brazil: $2784-$8755 / Q South Africa: $1367-$5783/Q  UK: $23535 /Q | Brazil:  DST vs TST: $2333.3/Q  DST vs IGRA: $840/ Q South Africa:  DST vs TST = $292.66 /Q DST vs IGRA = $9201.43/Q UK:  DST vs TST = $2089.17/ Q DST vs IGRA = $593.98/ Q |
| Chitpim et al. (20) | 1. SM + Culture and DST | 2: SSM + Xpert 3: SSM + TB-Lamb/DST 4: Xpert 5: TB-Lamb | VM: NA  M: NA  DS: Published literature from Thailand and other countries | QALYs | WTP threshold at 160,000 Baht per QALY | Almost all are cost-effective but option 5 dominant! |
| Brümmer et al. (44) | 1. No case finding | 2.Xpert Ultra 3.CRP-POC test: (65% sens. & 84% spec. reference Xpert) + Hypothetical scenarios | VM: NA  M: NA  DS: Published literature | DALYs, 1.9 / TP case detection  No incremental DALYs averted TB was not detected (i.e., FN) and no incremental DALYs accrued due to FP treatment but did incorporate FP treatment costs. Xpert Ultra used as reference for accuracy analysis | I: $560  P: $1061 SA: $3725 U: $192  V: $712  Societal opportun. cost-based | Strategy 1. vs 2.:  P: $670/D, V: $2000/D,  I: $1500 /D, SA: $1500/D, U: $1600/D  Strategy 1. vs 3.:  P: $550/D, V: $1500/D,  I: $1300/D, SA: $1300/D, U: $1200/D |
